# Potassium Disorders in Pet Rabbits and Their Association with Glycemia, Azotemia, and Clinical Outcome

**DOI:** 10.3390/ani16091372

**Published:** 2026-04-29

**Authors:** Maria Ardiaca, Daniel Pinto, Cristina Bonvehí, Andrés Montesinos

**Affiliations:** 1 Hospital Veterinario Medivet 24h Los Sauces, C/Santa Engracia 63, 28010 Madrid, Spain; cris.kbn@gmail.com (C.B.); amontesinosbarcelo@gmail.com (A.M.); 2 Faculdade de Medicina Veterinária, Universidade de Lisboa, Av. Universidade Técnica, 1300-477 Lisbon, Portugal; daniel.vet.pinto@gmail.com

**Keywords:** potassium, electrolyte, kalemia, rabbits, renal disease, azotemia, glucose, mortality risk

## Abstract

Potassium homeostasis is essential for maintaining membrane potential and normal neuromuscular function. Although potassium disturbances are clinically relevant in several species, their prevalence and prognostic significance in pet rabbits remain poorly characterized. This retrospective study evaluated plasma potassium concentrations at admission in 1773 venous samples from 1312 pet rabbits and assessed associations with mortality, glycemia, and renal markers (BUN and creatinine) using an i-Stat portable analyzer. Normokalemia (3.4–5.7 mmol/L) was observed in 78.1% of samples, while hypokalemia and hyperkalemia occurred in 13.9% and 8.0%, respectively. Overall, 7-day mortality was 21.3%, with most deaths (68.7%) occurring within 48 h. Both hypo- and hyperkalemia were associated with increased mortality, with hyperkalemia conferring the greatest risk (relative risk of up to 5.4 at 24 h; *p* < 0.0001). Potassium concentrations were higher in non-survivors at all time points. Hyperkalemia was also associated with hypoglycemia and azotemia (*p* < 0.0001), suggesting impaired renal potassium excretion and possible alterations in insulin-mediated cellular potassium uptake. No consistent association was observed between hyperglycemia and hyperkalemia. These findings indicate that plasma potassium disturbances are common in pet rabbits and are associated with short-term mortality and metabolic derangements. Early identification and correction of potassium imbalances and their causes may improve outcomes in critically ill rabbits.

## Simple Summary

Potassium is an essential mineral that helps control nerve signals, muscle contraction, and heart activity. Hence, abnormal potassium levels in the blood can be dangerous. Pet rabbits often arrive at veterinary hospitals with serious illnesses, yet the importance of changes in blood potassium levels in this species is not well understood. This study examined the laboratory results from 1773 blood samples collected from 1312 sick pet rabbits admitted to a veterinary hospital over an eleven-year period. The aim was to determine how often potassium disturbances occur and whether they are associated with survival, blood sugar, and kidney function. Most rabbits had potassium levels within the normal range, but about 22% showed abnormal values. Low potassium levels were more common than high potassium levels. Both conditions were associated with a higher risk of death, although rabbits with high potassium levels had the greatest risk. Nearly 70% of deaths occurred within the first two days of hospitalization. High potassium levels were also frequently found in the rabbits with low blood sugar and signs of impaired kidney function. These findings show that measuring potassium in rabbits at hospital admission can help veterinarians identify high risk patients and guide early treatment decisions that may improve survival.

## 1. Introduction

Potassium (K^+^) plays a crucial role in numerous cellular processes in vertebrates. The difference in K^+^ concentration between the intra- and extracellular compartments establishes the cell membrane potential, which is essential for functions such as nerve conduction and muscle contraction. This role cannot be compensated by other cations, making potassium homeostasis critical [1,2,3]. In humans, dogs, cats, horses, and ruminants, alterations in extracellular K^+^ disrupt the concentration gradient across cell membranes, affecting membrane polarization and potentially leading to clinically significant, life-threatening disorders that require prompt therapeutic intervention [1,2,3,4,5,6,7].

Analytical methods for assessing K^+^ balance are based on measuring extracellular K^+^ concentrations, typically in plasma [1,8]. Disturbances in plasma K^+^ can result from alterations in intake–excretion balance or from shifts between the intracellular and extracellular compartments. Hypokalemia is most commonly caused by low dietary intake, increased losses (e.g., diarrhea, renal losses), iatrogenic dilution of the intravascular compartment (e.g., intravenous fluids low in K^+^), or translocation of K^+^ into cells (as seen with metabolic alkalosis, bicarbonate administration, total parenteral nutrition, insulin-mediated responses, or excessive exogenous or endogenous corticosteroids [cortisol, corticosterone] and mineralocorticoids [aldosterone]). Rabbits are incapable of vomiting, so emesis is not a contributing factor to hypokalemia in this species. In cows, hypokalemia has been associated with intestinal ileus [9]. Hyperkalemia may result from excessive dietary intake, iatrogenic causes (e.g., potassium-containing drugs or excessive intravenous supplementation), reduced excretion, or translocation of K^+^ from the intracellular to the extracellular compartment. Because K^+^ excretion is primarily renal and regulated by adrenal function, kidney and adrenal disorders play a particularly important role in the development of spontaneous hyperkalemia [1,2,3,5,7,8,10].

In rabbits, hyperglycemia has been reported in association with severe stress, pain, and clinical conditions with poor prognosis, particularly intestinal obstruction [11]. Glucose metabolism in rabbits is primarily regulated by insulin, but the precise mechanisms underlying hyperglycemia in these patients remain unclear. In human medicine, insulin resistance induced by cortisol and inflammatory mediators is thought to contribute to hyperglycemia in critically ill patients [12,13]. Potassium metabolism is also partially regulated by insulin, which promotes K^+^ translocation into the intracellular compartment, thereby lowering plasma K^+^ concentrations. However, potassium disturbances and their relationship with glycemic disorders have not previously been evaluated in rabbits.

Although potassium homeostasis has been investigated in rabbits, spontaneous plasma potassium disorders and their clinical significance in pet rabbits have been minimally described to date [10,14,15,16,17,18,19]. The aim of this retrospective clinical study was to analyze the laboratory data of rabbit patients seen at our veterinary hospital, with a particular focus on plasma potassium concentrations, the prevalence of potassium disturbances and their association with mortality. Additionally, we sought to evaluate the relationship between potassium disturbances, glucose concentrations, and common markers of renal function (blood urea nitrogen (BUN) and creatinine). We hypothesized that pet rabbits exhibit plasma potassium disturbances, including hypo- and hyperkalemia, and that these disturbances are associated with glucose metabolism disorders, renal dysfunction, and an increased risk of mortality.

## 2. Materials and Methods

A retrospective study was conducted by reviewing the laboratory results and clinical records of clinically ill pet rabbits admitted between January 2015 and January 2026 at the Veterinary Hospital Medivet 24H Los Sauces (formerly Centro Veterinario Los Sauces from 2015 to 2021). Laboratory analyses were performed as part of the patients’ initial diagnostic workup, and all results were archived in annual Microsoft^®^ Excel spreadsheets. The recorded analytical data were screened to include only those from rabbits (*Oryctolagus cuniculus*). Clinical records were archived and reviewed using Winvet^®^ (Quality Compusoft S.L., Madrid, Spain) (2015–2022), Gestorvet^®^ (Softy Factory Solutions S.L., Las Palmas de Gran Canaria, Spain) (2022–2024), and Qvet^®^ (Q-Soft Tecnologías de la Información, S.L., Lleida, Spain) (2024–2026) software. Only blood samples collected at admission were included; follow-up analyses were excluded. For rabbits presented multiple times during the study period, inclusion required a minimum interval of four months since the previous visit. Rabbits receiving any medication at the time of presentation were excluded to minimize potential drug-related effects on plasma K^+^ levels.

Extracted data included sex; age; sample quality (assessed visually for hemolysis or lipemia); plasma concentrations of K^+^, glucose, blood urea nitrogen (BUN), and creatinine; date of presentation; and date of demise (if applicable). Mortality was evaluated at 24 h, 48 h, and within 7 days of admission. Rabbits were grouped accordingly (Survivors-24 h, Non-survivors-24 h, Survivors-48 h, Non-survivors-48 h, Survivors-7 d, Non-survivors-7 d). Rabbits with unknown outcomes were excluded. Animals that were euthanized or died following surgical procedures were also excluded from mortality analyses. Samples showing hemolysis or lipemia were excluded.

The prevalence of potassium disturbances and their associations with glucose, BUN, creatinine, and outcomes were evaluated. Previously established reference ranges using the same analytical methods were applied for interpretation [16,20]. Normokalemia was defined as plasma K^+^ of 3.4–5.7 mmol/L. Hypokalemia and hyperkalemia were defined as values below or above this range, respectively. Azotemia was defined as BUN > 12.5 mmol/L and/or creatinine > 221 µmol/L [16,21]. Rabbits were classified as non-azotemic only when both values were within normal limits. Normoglycemia was defined as plasma glucose of 5.2–13.6 mmol/L [16]. Hypoglycemia and hyperglycemia were defined as values below or above this range, respectively.

All samples were collected in lithium heparin from the lateral saphenous vein using previously described techniques [16,22]. Samples were processed within 2 min. Direct ion-selective electrode methods were used with the i-Stat One portable analyzer (Abaxis Inc., (Union City, CA, USA) Abbott Point of Care (Princeton, NJ, USA) from 2015 to 2021, and the i-Stat Alinity V portable analyzer (Zoetis España S.L., (Madrid, Spain), Abbott Point of Care) from 2022 to 2026. Disposable cartridges (i-Stat EC8+, CG8+, and Chem8+) were equilibrated to room temperature before use.

Statistical analyses were performed using MedCalc Statistical Software version 20.021 (64-bit). Extreme values were capped for calculations. K^+^ > 9 mmol/L was set to 9 mmol/L, and K^+^ < 2 mmol/L was set to 2 mmol/L. BUN > 50.9 mmol/L was set to 50.9 mmol/L. Glucose < 1.1 mmol/L was set to 1.1 mmol/L, and glucose > 38.9 mmol/L was set to 38.9 mmol/L. Normality was assessed using the Kolmogorov–Smirnov test. Outliers were identified using Tukey’s method but were retained, as they represented valid clinical data. Non-parametric tests (Mann–Whitney U and Kruskal–Wallis) were used for group comparisons. The Chi-squared test was used to assess relationships between categorical variables. In order to evaluate the strength of the association of the K levels and outcome, logistic regression analysis was performed assessing the relationship between the outcome at 24 h, 48 h and 7 days with potassium disturbances (hyperkalemia, hypokalemia), azotemia (azotemic, non-azotemic) and glycemia (hypoglycemia, hyperglycemia) in a multivariable model.

## 3. Results

### 3.1. Patients

A total of 1773 venous samples from 1312 pet rabbits (735 males, 569 females, and 8 with unregistered sex) collected between January 2015 and February 2026 met the inclusion criteria. A total of 267 rabbits were presented more than once during the study period. These rabbits contributed multiple samples as follows: 2 samples (*n* = 159), 3 samples (*n* = 56), 4 samples (*n* = 32), 5 samples (*n* = 9), 6 samples (*n* = 7), 7 samples (*n* = 3), and 10 samples (*n* = 1).

**Figure 1:**
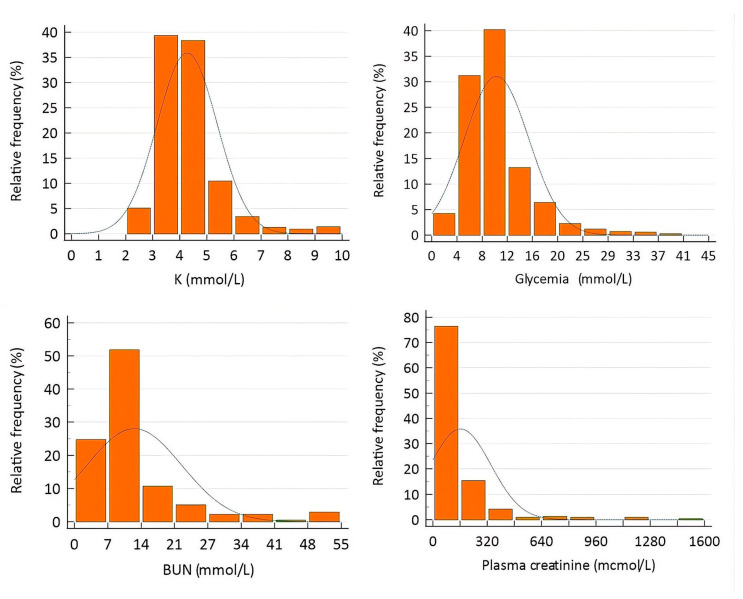
Histogram of on-arrival plasma concentrations of potassium (K), glucose (Glu), blood urea nitrogen (BUN), and creatinine in pet rabbits. The line represents the Normal distribution. Data distribution was non-normal for all parameters (Kolmogorov–Smirnov test, *p* < 0.0001).

K^+^ concentrations were determined in all samples. Concurrent measurements were available for glucose (*n* = 1649), BUN (*n* = 1382), and creatinine (*n* = 271). No statistically significant differences were observed between males and females in plasma concentrations of potassium, glucose, or BUN (Kruskal–Wallis; *p* = 0.74, 0.42, and 0.56, respectively). In contrast, creatinine concentrations differed significantly between sexes (Kruskal–Wallis; *p* = 0.026). Median creatinine values were higher in males (114.9 µmol/L) than in females (106.8 µmol/L). Descriptive statistics for these data are presented in Table 1 and Figure 1.

### 3.2. Prevalence of Potassium, Glycemia, BUN and Creatinine Disturbances

Of the 1773 rabbits, 1385 (78.1%) had normokalemia. Hypokalemia was present in 247 rabbits (13.9%), and hyperkalemia in 141 rabbits (8.0%). Regarding glycemia, 1225 rabbits (74.3%) were normoglycemic. Hypoglycemia was observed in 107 rabbits (6.5%), and hyperglycemia in 317 rabbits (19.2%). Elevated BUN concentrations were found in 419 rabbits (30.3%). Elevated creatinine concentrations were observed in 52 rabbits (19.2%). Overall, azotemia was present in 431 rabbits. The absence of azotemia was confirmed in 185 rabbits.

### 3.3. Potassium Disturbances and Mortality

**Figure 2:**
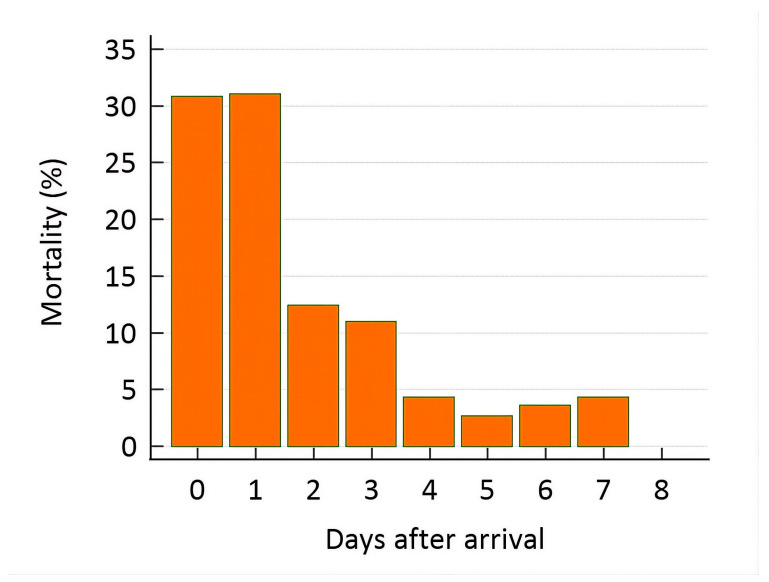
Daily mortality rate during the first 7 days after admission.

A total of 419 of 1773 rabbits (23.6%) died within 7 days of admission. In contrast, 1349 rabbits survived. Outcome data were unavailable for 5 animals. Thirteen rabbits died following surgery, and 29 were euthanized. These cases were excluded from the mortality analyses. After these exclusions, 377 rabbits (21.3%) were classified as non-survivors at 7 days (Non-survivors-7 d). Among these, 129 rabbits (34.2%) died within the first 24 h (Non-survivors-24 h), and 259 of 377 rabbits (68.7%) died within the first 48 h (Non-survivors-48 h). Mortality rates decreased over the following days (Figure 2). Mortality at all time points (24 h, 48 h, and 7 days) did not differ between males and females (chi-square test, *p* > 0.05).

**Figure 3:**
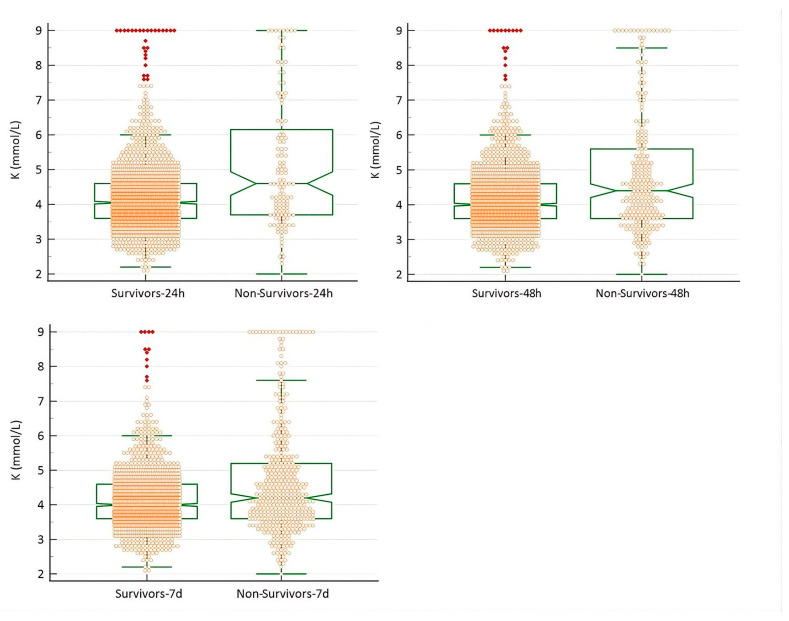
Notched box-and-whisker plots representing potassium (K) concentrations in rabbits (*n* = 1773) in relation to the outcome at 24 h, 48 h and 7 days after admission. Potassium concentrations were higher in the non-survivor groups at all time points (Mann–Whitney U; *p* < 0.0001).

At each time point (24 h, 48 h, and 7 days), K^+^ levels were compared between survivors and non-survivors. Potassium concentrations were higher in the non-survivor groups (Mann–Whitney U; *p* < 0.0001). However, there was considerable overlap between groups (Figure 3).

**Figure 4:**
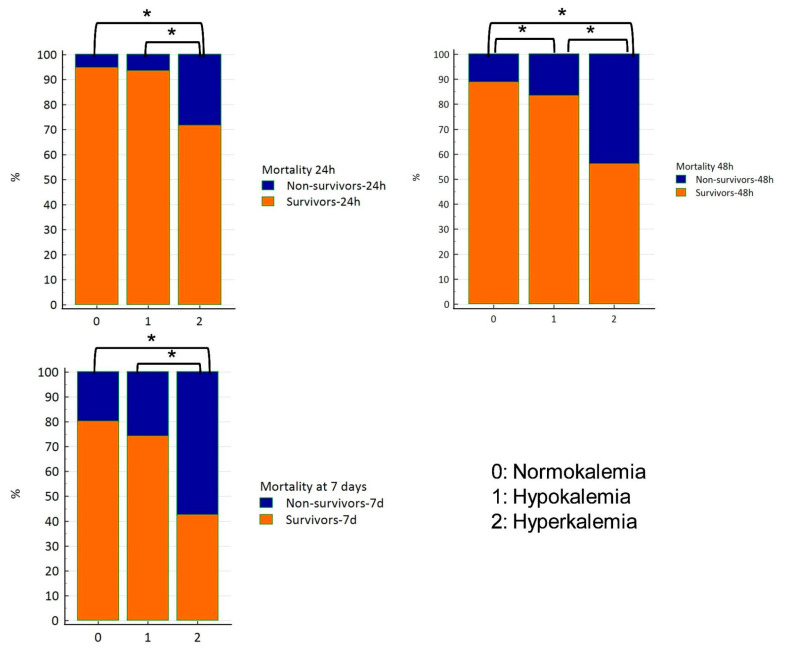
Stacked column frequency charts representing the outcome in terms of survival at 24 h, 48 h and 7 days in the rabbits presenting normo-, hypo-, or hyperkalemia on admission. Differences between groups are indicated by brackets. Black brackets with an asterisk (*) denote significance (chi-squared test; *p* < 0.0001).

Significant differences in mortality rates were observed among normokalemic, hypokalemic, and hyperkalemic rabbits at 24 h, 48 h, and 7 days post-admission (chi-squared test; *p* < 0.0001) (Figure 4). Hyperkalemic rabbits had a higher risk of mortality than both normokalemic and hypokalemic rabbits (Table 2). Logistic regression analysis examining the association between outcomes and potassium disturbances, azotemia, and glycemia in a multivariable model showed that hyperkalemia, but not hypokalemia, was significantly associated with outcomes at 24 h, 48 h, and 7 days (*p* < 0.0001).

### 3.4. Potassium Disturbances and Their Association with Glycemia and Renal Disease Markers (BUN and Creatinine)

**Figure 5:**
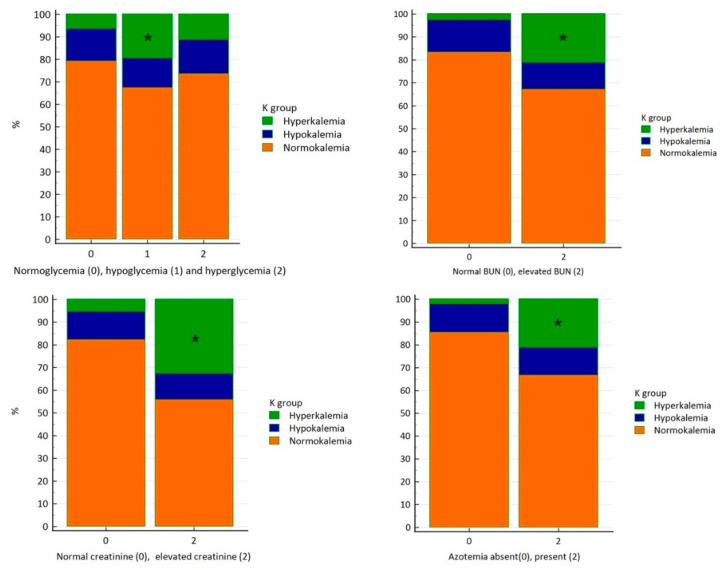
Stacked column frequency charts showing the prevalence of normokalemia, hypokalemia, and hyperkalemia in rabbits in relation to plasma imbalances of glucose, BUN, and creatinine. Bars marked with an asterisk (*) indicate statistically significant differences between groups. Hyperkalemia was more common in hypoglycemic rabbits and in rabbits with elevated BUN or creatinine (Kruskal–Wallis, *p* < 0.005; chi-squared, *p* < 0.0001).

Potassium imbalances were associated with changes in plasma glucose, BUN, and creatinine. Hyperkalemia was more common in hypoglycemic rabbits than in normoglycemic rabbits (Kruskal–Wallis, *p* = 0.039; chi-squared, *p* < 0.0001). It was also more frequent in rabbits with elevated BUN than in those with normal BUN (Kruskal–Wallis, *p* < 0.0001; chi-squared, *p* < 0.0001). Similarly, hyperkalemia was more common in rabbits with elevated creatinine than in those with normal creatinine (Kruskal–Wallis, *p* < 0.0001; chi-squared, *p* < 0.0001). It was also more frequent in rabbits with azotemia than in non-azotemic rabbits (Kruskal–Wallis, *p* < 0.0001; chi-squared, *p* < 0.0001) (Figure 5).

## 4. Discussion

The overall prevalence of abnormal potassium concentrations in pet rabbits was 21.9%, with hypokalemia (13.9%) occurring more frequently than hyperkalemia (8%). This prevalence is lower than that reported in dogs, cats, and horses [3,23]. Consistent with our initial hypothesis, both hypokalemia and hyperkalemia were associated with an increased risk of mortality. Hyperkalemia appeared to be particularly life-threatening, whereas hypokalemia, although clinically relevant, had a more moderate impact on mortality risk in this study. There was considerable overlap in potassium concentrations between survivors and non-survivors. (Figure 3) As previously reported in other species, although potassium imbalances are associated with increased mortality, they are not necessarily reliable prognostic indicators. In clinical practice, outcomes reflect the complex interplay of patient-specific pathological processes and are not determined by analytical parameters alone, but also by other factors such as adequacy of treatment and individual resilience. However, when identified, potassium disturbances warrant prompt recognition and appropriate therapeutic adjustment [1,4]. Notably, most rabbits (68.7%) admitted to our hospital that did not survive died within the first 48 h, underscoring the importance of early diagnosis and timely therapeutic intervention in critically ill patients.

Potassium disturbances were also associated with alterations in glycemia and renal function markers (BUN and creatinine). Hyperkalemia was most frequently observed in hypoglycemic rabbits. This finding is consistent with the expected reduction in plasma insulin concentrations during hypoglycemia. Lower insulin levels decrease potassium translocation into the intracellular compartment, thereby increasing the risk of hyperkalemia. No association was identified between hyperglycemia and hyperkalemia in rabbits in our study. A concurrent occurrence of hyperglycemia and hyperkalemia would be expected primarily in cases of insulin resistance, which is one proposed mechanism for hyperglycemia in critically ill human patients [12,13]. Although hyperglycemia is a common finding in ill rabbits, the lack of a statistical association with hyperkalemia suggests that insulin resistance is not the primary underlying mechanism in this species. However, some rabbits did exhibit concurrent hyperglycemia and hyperkalemia, indicating that insulin resistance or impaired cellular potassium uptake may occur in a subset of cases. Hyperglycemia is particularly frequent in rabbits with intestinal obstruction. Interestingly, in dogs with intestinal obstruction, hypokalemia is one of the most common findings [24]. Hypokalemia has also been reported in 23.2% of rabbits presenting with acute gastric dilatation [17]. In this study, hyperglycemia was not consistently associated with hypokalemia in rabbits.

In other species, severe hyperkalemia most commonly results from renal or adrenal dysfunction [1,4,8,10,23]. In the rabbits included in this study, altered potassium concentrations were likewise associated with disturbances in renal function markers. In particular, hyperkalemia was most frequently observed in animals with evidence of impaired renal function. However, as reported in other species, the association between hyperkalemia and renal failure in rabbits is neither absolute nor consistent [1,3]. Hyperkalemia may also arise from other conditions, including uroperitoneum, severe dehydration, or acidosis [1,4,8,10,23]. Therefore, potassium concentration cannot be inferred solely from the renal profile on blood chemistry, and direct measurement of potassium concentration is required for accurate diagnostic assessment in each individual patient. Sample quality must be carefully assessed to ensure the reliability and accuracy of the results. Flame photometry and ion-selective electrode (such as the method used in the present study) methods are preferred to optical methods [25].

In humans, dogs, cats, and horses, Addison’s disease (hypoadrenocorticism) is relatively common, is frequently associated with hyperkalemia, and can be misdiagnosed as renal failure [1,26]. In contrast, spontaneous adrenal gland disease in pet rabbits—primarily hyperplasia and neoplasia—is rare. When reported, it is most associated with elevated sex hormone levels and has not been shown to affect potassium concentrations in this species [27,28]. Several rabbits in this study presented findings compatible with hypoadrenocorticism (hyperkalemia with concomitant hypoglycemia and azotemia). Future studies focused on contrasting clinical and laboratory findings may elucidate the etiologies of potassium disturbances in pet rabbits.

Slight differences in creatinine values between males and females observed in our study can be attributed to an artifact due to the relatively small sample size (*n* = 269), a greater muscular mass in male rabbits, or lower capacity of recovery from renal injury in males, as has been shown in other mammalian species [29,30,31,32]. The clinical relevance of this finding remains unclear and is likely limited.

This study focused on acute disturbances in potassium concentrations. The patients included in this study were clinically ill rabbits. Particular care was taken to minimize the risk of including chronic cases. At our hospital, blood gas and electrolyte analyses are performed only in cases of clinical illness and are not routinely conducted during wellness examinations or elective surgical procedures. In addition, rabbits receiving medication at the time of presentation were excluded. Potassium levels typically fluctuate within a physiological range across patients and reflect the acute clinical condition rather than inherent patient-specific traits. A minimum interval of four months since the previous visit was considered sufficient to treat each presentation as an independent event, thereby limiting potential bias from repeated measures in individual patients.

The main limitations of this study are its retrospective, single-center design and the lack of data linking potassium abnormalities to specific clinical diagnoses. Rabbits often present with subtle and nonspecific clinical signs, and many of the included cases may have involved multiple concurrent diseases, complicating clinical classification. Despite clinicians’ best efforts, including thorough examination and diagnostic testing, diagnoses—in the absence of established consensus guidelines and standardized protocols—still rely heavily on individual interpretation. Inevitably, this introduces variability in clinical judgment and reduces data reliability. In this study, markers of renal dysfunction (elevated BUN, elevated creatinine, or both) and alterations in glycemia were considered more objective and consistent indicators than clinical diagnoses. Future studies evaluating potassium abnormalities in rabbits with specific pathologies defined by well-established diagnostic criteria are warranted to better clarify the clinical implications of potassium disorders in this species.

## 5. Conclusions

Plasma potassium disturbances were found to be relatively common in the pet rabbit population in this study, although their prevalence (21.9%) is lower than that reported in dogs, cats, and horses. Hypokalemia (13.9%) was more frequent than hyperkalemia (8.0%). Both conditions were associated with increased short-term mortality; however, hyperkalemia carried a markedly higher risk of death, particularly within the first 24–48 h after admission. Most mortality events occurred early, with nearly 70% of deaths taking place within 48 h, highlighting the critical importance of rapid assessment and intervention at presentation. There was considerable overlap in potassium concentrations between survivors and non-survivors. At each time point, both groups spanned the full range of K^+^ levels (from <2 mmol/L to >9 mmol/L) (Figure 3). This indicates that, although potassium disturbances represent a risk factor, individual potassium values alone are not fully predictive of the outcome. Hyperkalemia was strongly associated with markers of renal dysfunction, including elevated BUN and creatinine, supporting impaired renal excretion as a major contributing factor. It was also significantly associated with hypoglycemia, suggesting a potential role for altered insulin-mediated potassium regulation in critically ill hypoglycemic rabbits. Notably, hyperkalemia could occur independently of renal disease and hypoglycemia, indicating that other conditions may contribute to elevated plasma potassium in these rabbits. This finding warrants further research comparing clinical and laboratory data to clarify the origins of potassium disturbances.

## Figures and Tables

**Table 1:** Summary statistics for the plasma concentrations of K^+^, glucose, BUN, and creatinine in pet rabbits.

	K^+^ (mmol/L)	Glucose (mmol/L)	BUN (mmol/L)	Crea (µmol/L)
N	1773	1649	1382	271
Minimum	2.0	0.83	1.1	17.7
Maximum	9.0	38.9	50.9	1644.2
Mean	4.3	10.6	13.8	205.3
Median	4.1	9.3	9.5	114.9
Standard deviation	1.11	5.55	11.53	265.45
Relative standard deviation	0.26	0.52	0.84	1.29
25–75 Percentiles	3.6 to 4.7	7.3 to 12.2	6.9 to 14.9	88.4 to 185.6
Kolmogorov–Smirnov test for Normal distribution (*p* value)	<0.0001	<0.0001	<0.0001	<0.0001
Outliers (Tukey test)				
N	50	74	81	12
Range	6.4–8.0	19.6–26.4	26.9–38.5	335.9–468.5
Far out values (Tukey test)				
N	38	40	87	22
Range	8.1–9.0	27.0–38.9	39.3–50.9	477.4–1644.2
N values under lower limit of detection	1 (<2 mmol/L)	20 (<1.1 mmol/L)	0	0
N values over upper limit of detection	11 (>9 mmol/L)	6 (>38.9 mmol/L)	59 (>50.9 mmol/L)	0

**Table 2:** Relative risk of mortality at 24 h, 48 h and 7 days and odds ratio in the rabbits presented with hypokalemia or hyperkalemia compared to the rabbits presented with normokalemia.

	Outcome 24 h	Outcome 48 h	Outcome 7 d
Hypokalemia			
Relative risk	1.2 (*p* = 0.4404)	1.5 (*p* = 0.0162) *	1.3 (*p* = 0.0519)
Odds ratio	1.2 (*p* = 0.4424)	1.6 (*p* = 0.0185) *	1.4 (*p* = 0.0574)
Hyperkalemia			
Relative risk	5.4 (*p* < 0.0001) *	3.9 (*p* < 0.0001) *	2.9 (*p* < 0.0001) *
Odds ratio	7.1 (*p* < 0.0001) *	6.2 (*p* < 0.0001) *	5.4 (*p* < 0.0001) *

* Statistically significant results.

## Data Availability

The original data presented in the study are openly available in Data potassium pet rabbits.xlsx spreadsheet at https://1drv.ms/x/c/a3e28fff7f42b2ec/IQBMpdrZQYkOQIfQxKHZipOxAaNfpqkoI_Iz3a6joPj40NE?e=eogYGT (accessed on 23 April 2026).
